# Greenspace, Air Pollution, Neighborhood Factors, and Preeclampsia in a Population-Based Case-Control Study in California

**DOI:** 10.3390/ijerph18105127

**Published:** 2021-05-12

**Authors:** Kari A. Weber, Wei Yang, Evan Lyons, David K. Stevenson, Amy M. Padula, Gary M. Shaw

**Affiliations:** 1 Department of Pediatrics, Division of Neonatal and Developmental Medicine, Stanford University School of Medicine, Stanford, CA 94305, USA; wyang3@stanford.edu (W.Y.); dks750@stanford.edu (D.K.S.); gmshaw@stanford.edu (G.M.S.); 2 Department of Earth System Science, Stanford University, Stanford, CA 94305, USA; elyons.geog@gmail.com; 3 Department of Obstetrics, Gynecology and Reproductive Sciences, University of California, San Francisco, CA 94143, USA; amy.padula@ucsf.edu

**Keywords:** greenspace, preeclampsia, air pollution, neighborhood, pregnancy

## Abstract

To investigate preeclampsia etiologies, we examined relationships between greenspace, air pollution, and neighborhood factors. Data were from hospital records and geocoded residences of 77,406 women in San Joaquin Valley, California from 2000 to 2006. Preeclampsia was divided into mild, severe, or superimposed onto pre-existing hypertension. Greenspace within 100 and 500 m residential buffers was estimated from satellite data using normalized difference vegetation index (NDVI). Air quality data were averaged over pregnancy from daily 24-h averages of nitrogen dioxide, particulate matter <10 µm (PM_10_) and <2.5 µm (PM_2.5_), and carbon monoxide. Neighborhood socioeconomic (SES) factors included living below the federal poverty level and median annual income using 2000 US Census data. Odds of preeclampsia were estimated using logistic regression. Effect modification was assessed using Wald tests. More greenspace (500 m) was inversely associated with superimposed preeclampsia (OR = 0.57). High PM_2.5_ and low SES were associated with mild and severe preeclampsia. We observed differences in associations between greenspace (500 m) and superimposed preeclampsia by neighborhood income and between greenspace (500 m) and severe preeclampsia by PM_10_, overall and among those living in higher SES neighborhoods. Less greenspace, high particulate matter, and high-poverty/low-income neighborhoods were associated with preeclampsia, and effect modification was observed between these exposures. Further research into exposure combinations and preeclampsia is warranted.

## 1. Introduction

Preeclampsia is a hypertensive disorder affecting 3–5% of pregnant women, and it is a major contributor to maternal and neonatal mortality [1,2]. Traditionally diagnosed as high blood pressure in combination with proteinuria after 20 weeks of pregnancy, there are few known risk factors [2]. The causes of preeclampsia remain unknown, but the underlying pathology is known to originate in the placenta during the first trimester, possibly from vascular compromise during abnormal placentation [3,4]. Exposure to various environmental factors had been shown to cause oxidative stress, possibly leading to developmental toxicity and affecting placental development [5].

To explore the potential etiologies of preeclampsia, in previous studies, we examined various environmental exposures and their associations with preeclampsia including residential proximity to greenspace [6]. Greenspace, including grass, trees, and other vegetation, has been positively associated with a variety of health outcomes [7,8]. In our study of greenspace, we observed an inverse association between living surrounded by a higher density of greenspace and preeclampsia superimposed onto pre-existing hypertension, a particular preeclampsia phenotype [6].

The myriad proposed beneficial mechanisms of greenspace include the abatement of air pollution, heat, and noise, stress reduction, and a greater opportunity for physical activity [9]. Some of these exposures have been associated with preeclampsia. Air pollutants have been associated with preeclampsia, and a meta-analysis found an increase in PM_2.5_ to be associated with a 30% increase in preeclampsia [10]. Other studies have observed associations between environmental noise and severe preeclampsia [11], perceived stress and preeclampsia [12], and physical activity and preeclampsia [13]. Exposure to greenspace as a direct effect (e.g., decreased air pollution) or a proximal effect (e.g., reduced stress or more physical activity) may be an important factor that could be modified to alter the population burden of preeclampsia. Additionally, these potential mechanisms may be modified by neighborhood socioeconomic factors that impact the quality and use of greenspaces.

Using various available data sources in a highly polluted and diverse area of California, we expanded our previous work to examine greenspace along with air pollution and neighborhood socioeconomic factors. We explored these exposures independently and jointly to determine potential excess benefit or risk due to co-exposure and to attempt to understand how residential proximity to greenspace during pregnancy may help to reduce the risk of preeclampsia.

## 2. Materials and Methods

This case-control study focused on 329,650 live births delivered in California between 2000 and 2006 from four counties in the San Joaquin Valley (Fresno, Kern, Stanislaus, and San Joaquin). Births not meeting eligibility criteria were excluded. To be included, births had to be singleton, have a weight within 500–5000 g, and be 20–41 weeks gestational age. Data from the Office of Statewide Health and Planning (OSHPD) maternal and infant discharge records were linked with California birth certificates. Linkage was successful in 99% of records using a previously described algorithm [14,15]. Data from this linkage included demographic data for the women and their infants, as well as clinical data from the infant’s delivery. Maternal residential addresses were obtained from birth certificates. Addresses were geocoded using the REST API Geocode Service from the California Department of Public Health Information Technology Services Division, resulting in the successful geocoding for 94% of records.

Data from this population have been utilized previously to study greenspace [6], pesticides [16], traffic-related air pollutants [17], preterm birth [18], and preeclampsia [16]. Detailed methods have been published elsewhere but are briefly described below.

There were 78,421 preterm births (<37 weeks of gestation) in this population included in our analytic base. Among the term births (>37 weeks of gestation), a randomly selected subset of 235,263 were included. To specifically investigate preeclampsia, women diagnosed with preeclampsia who delivered at term were excluded (2% of the controls). Diagnoses of preeclampsia were identified using *the International Classification of Diseases, 9th Revision, Clinical Modification* (ICD-9-CM) codes. Women with preeclampsia were classified as having mild preeclampsia (642.4), severe preeclampsia/eclampsia (642.5 and 642.6), and preeclampsia or eclampsia superimposed onto pre-existing hypertension (642.7). To create mutually exclusive groups, women were reclassified. Those with a code for pre-existing hypertension (401–405, 642.0, 642.1, 642.2, and 642.9) and one of the preeclampsia codes were reclassified as having preeclampsia superimposed onto pre-existing hypertension. Women with multiple preeclampsia codes were reclassified to the most severe condition.

Greenspace was estimated as the normalized difference vegetation index (NDVI). NDVI is the normalized difference between visible and near-infrared region (NIR) wavelengths reflected by the Earth’s surface. Ranging from −1 to 1, with values closer to 1 corresponding to a higher density of green [19].

NDVI =(NIR−Red)/(NIR + Red)

The full specifications were described elsewhere [6]. Briefly, we created maximum NDVI Landsat images at a 30 m spatial resolution using Google Earth Engine [20]. Using the “greenest” time of that year, peak vegetation with no cloud cover, we obtained a maximum NDVI. The average maximum NDVI was separately estimated for each year, and women were assigned NDVI values for the year of the estimated conception of the infant.

We linked each Google Earth Engine file with the geocoded maternal addresses for each birth in ArcGIS (ESRI, Release 10.6.1. Redlands, CA, USA). The average NDVI, our estimation of “greenness,” was calculated within 100 and 500 m concentric buffers around each maternal residence. Multiple buffers were used based on hypothesized differences in greenspace mechanisms. For example, if there is a large park or forest further than 100 m away but within the 500 m buffer with little greenness very close to the woman’s home, the 500 m buffer NDVI may be much higher than the 100 m buffer NDVI. USGS considers a high NDVI to be 0.6–0.9, which would be roughly a forest at its greenest point.

Air quality data were obtained from U.S. Environmental Protection Agency’s Air Quality System database during the same time period (https://aqs.epa.gov/aqsweb/documents/data_mart_welcome.html, accessed on 1 May 2008). Daily 24-h averages of nitrogen dioxide (NO_2_), particulate matter <10 µm (PM_10_), particulate matter <2.5 µm (PM_2.5_), and carbon monoxide (CO) were collected. Inverse distance-squared weighting was used to interpolate air quality using up to four air quality measurement stations. A maximum interpolation radius of 50 km for NO_2,_ PM_10_, and PM_2.5_ was used and 25 km for CO. Residences located within 5 km of 1 or more monitoring stations used values that were interpolated based solely on those stations. Averages were calculated over the entire pregnancy. Records with at least one exposure to any of the four pollutants were included. A “cumulative” pollutant score was also derived for participants with data on at least three pollutants. Each pollutant was coded as 0 in the lower 3 quartiles and 1 in the highest quartile. The pollutants were then summed, ranging from 0–4, so those who were assigned within the lower 3 quartiles for all exposures had a score of 0 and those in the highest quartile of exposure for all exposures received a score of 4. We categorized the pollutant score into two levels, i.e., a score of >1 was coded as 1 (about 27%) and a score of ≤1 was coded as 0 (about 77%).

Neighborhood socioeconomic (SES) factors were derived from the 2000 US Census data. They included residing in a census block, with more than 20% living below the federal poverty level (high poverty) and residing in a block with a median annual income of less than $30,000 (low income).

Records with both greenspace and air pollution data resulted in 77,406 births. Distributions of participant characteristics were determined among cases by preeclampsia phenotype and controls. There were no major differences between the demographic distributions of the subset compared to the original greenspace sample. For each buffer (100 and 500 m), the quartiles of average NDVIs were determined using cutoff values among controls. Correlations among exposures of interest (i.e., greenspace within each buffer, air pollutants, and neighborhood SES variables) were calculated using Pearson correlation coefficients. Odds ratios (ORs) and 95% confidence intervals (95% CIs) were estimated using logistic regression. They were calculated by comparing women surrounded by the highest to the lowest quartiles of greenspace for each preeclampsia phenotype. ORs and 95% CIs were also calculated by comparing women exposed to the highest quartile of each pollutant to those in the lower three quartiles (to be consistent with previous work in this population [21]) and women in the high-poverty and low-income groups compared to those not in each group for each preeclampsia phenotype. Analyses were adjusted for maternal age, race/ethnicity, and season of conception (winter, spring, summer, and fall). These variables were available from OSHPD and defined as potential confounders due to an observed association between preeclampsia and exposure.

To explore potential reductions in risk associated with greenspace, we performed effect modification and mediation analyses with other hypothesized interrelated exposures. In the effect modification analyses, ORs and 95% CIs were estimated to assess the association between residing in a high density of greenspace within a 500 m buffer, and each preeclampsia phenotype modified by a high exposure to PM_10_ and PM_2.5_, as well as by the neighborhood SES variables. Interaction was assessed using Wald’s chi-squared test. Estimates stratified by PM_10_ and PM_2.5_ were calculated overall and further stratified by neighborhood SES factors. Exposure to particulate matter was also analyzed as a mediator between greenspace and preeclampsia. Using multivariable logistic regression, direct and indirect effects of greenspace were estimated for each preeclampsia phenotype and the percentage mediated by PM_10_ and PM_2.5_ [22,23].

All analyses were performed using SAS version 9.4 (SAS Institute, Cary, NC, USA). This project employed data from State of California databases. The use of these data was approved by both the State Committee for the Protection of Human Subjects, as well as Stanford University IRB (24543).

## 3. Results

Compared to control women, women with superimposed preeclampsia were more likely to be older or Black. Mild or severe cases of preeclampsia were more likely to be younger than 20 years of age, US-born Hispanic, and nulliparous (Table 1). There were few differences observed between women in the highest quartile of greenspace in the 100 or 500 m residential buffers compared to the whole sample, except that women residing in the highest quartiles of greenspace were more likely to live in a low-poverty or high-income census block (Table 2).

Among women in the control group, residential greenspace within 100 and 500 m buffers were moderately correlated with each other but were not strongly correlated with the other exposures, and PM_2.5_ was moderately correlated with the other air pollutants (Supplementary Table S1).

Residing in the highest density of greenspace (within a 500 m buffer) was inversely associated with superimposed preeclampsia (aOR: 0.56; 95% CI: 0.40–0.80) and there were suggestive inverse associations with mild preeclampsia for both buffers (within 100 and 500 m) (Table 3). Higher exposure to PM_2.5_ showed an association for mild (aOR: 1.28; 95% CI: 1.10–1.49) and severe preeclampsia (aOR: 1.38; 95% CI: 1.19–1.59), and there was a suggestive association with superimposed preeclampsia. Low neighborhood SES showed an association with each preeclampsia phenotype, although the confidence interval for the OR for superimposed preeclampsia included 1.0 (Table 3).

To explore the interrelatedness of greenspace, air pollution, and neighborhood SES, we performed effect modification analyses. For neighborhood SES, we did not observe heterogeneity between the neighborhood SES variables for mild or severe preeclampsia. We did observe heterogeneity by neighborhood income for women with superimposed preeclampsia; associations were inverse amongst those in the low-poverty and high-income groups, and they were null or moderately inverse in the low-income and high-poverty groups, respectively (Table 4). Statistical evidence of heterogeneity was observed for greenspace and severe preeclampsia associations between the high and low PM_10_ exposure categories (overall and specifically those women in the high-income category). For these heterogeneous associations, estimates for the high PM_10_ strata tended toward higher odds, and estimates for the low PM_10_ strata were generally null (Table 5). Among those in high-poverty or low-income neighborhoods, there was little to no heterogeneity by level of pollutant.

**Figure 1:**
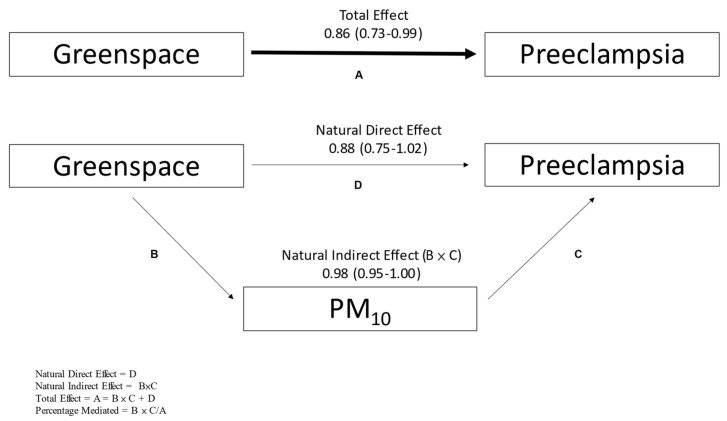
An example of the procedure used for mediation analyses. In this example, PM_10_ mediated the association between greenspace and mild preeclampsia by 15%.

Overall, we did not observe significant mediation by air pollutants (results not shown). A suggestive association was observed for mediation by PM_10_, and an example of the procedure and results are displayed in Figure 1. The association between greenspace (100 m buffer) and mild preeclampsia was 15% mediated by PM_10_, although it did not reach statistical significance.

## 4. Discussion

This study sought to examine the potential interdependencies between greenspace, traffic-related air pollution, and neighborhood socioeconomic factors for risk of preeclampsia in a diverse and highly polluted area of California. We previously observed an inverse association between women residing near a higher density of greenspace during pregnancy and superimposed preeclampsia [6]. We attempted to extend these findings to other aspects of women’s environment and social context. We hypothesized that any potential effects of greenspace would be modified or mediated by additional environmental factors.

The two additional environmental factors we explored were air pollution and neighborhood SES. As expected, we observed associations between these factors, specifically PM_2.5_, PM_10_, and SES, with the preeclampsia phenotypes. Contrary to what we expected, we did not observe meaningful mediation in the associations between greenspace and preeclampsia by other exposures.

In our effect modification analyses, we did observe heterogeneity between the associations of greenspace and superimposed preeclampsia after stratification by neighborhood SES, though not in the direction we expected. After stratification by level of PM_2.5_ and PM_10_, the patterns we observed for associations with superimposed preeclampsia remained similar among those exposed to both high and low levels of particulate matter. These results suggested that the observed reductions in risk observed between greenspace and superimposed preeclampsia were stronger among those residing in higher SES neighborhoods but independent of air pollution.

Given that the observed statistical heterogeneity was observed among people living in high-income or low-poverty neighborhoods, greenspace in these areas may reflect something different than a high greenspace area in a lower SES neighborhood. For example, the greenspaces in lower SES neighborhoods may be of lower quality and not utilized in the same ways as those in higher SES neighborhoods. One study found that greenspaces in lower SES neighborhoods had more safety concerns and fewer amenities like restrooms, equipment, and seating [24]. Such disparities in greenspace quality may have accounted for the differences observed in our study, though more study is required in this area.

For severe preeclampsia, we observed heterogeneity after stratification by particulate matter, with suggestive positive associations for women exposed to high levels of PM_10_. There are many possible conclusions to be drawn from these results. A recent review found that in most studies of greenspace and air pollutants, greenspace lessened the surrounding air pollution, especially parks, and tree canopy [25]. Thus, given their interdependencies, it may not be possible to fully disentangle the effects of air pollution and greenspace. Among those with exposure to higher levels of PM_10_ and PM_2.5_, the results were counterintuitive, with greenspace being positively associated with severe preeclampsia. Again, given the lack of specific data regarding the type or utilization of greenspace in this population, more study is warranted.

The benefits of greenspace have been documented for a wide variety of health benefits [7]. To our knowledge, there has only been one other study of greenspace and preeclampsia, and it did not observe an association [26]. However, that study assessed preeclampsia as a single outcome, unlike our assessment of specific phenotypes, and the largest residential buffer used in that study was 150 m; meanwhile, the inverse associations in our population were observed for greenspace within a 500 m buffer. To the best of our knowledge, ours was the first study to assess potential effect modification by high levels of PM_2.5_ and PM_10_. Previous studies of greenspace and birth outcomes, predominantly birthweight and preterm birth, have explored potential differences by socioeconomic status with inconsistent results [27,28,29,30]. Direct comparison with these studies is not possible because the measured outcomes and the variables used to delineate SES were different.

This study had many strengths and some limitations. One limitation of this study was that our method of greenspace estimation, NDVI, did not allow us to distinguish different types of greenspaces. Depending on the method of potential greenspace benefits, different types could yield different results (e.g., urban tree canopy reducing pollution versus a park that is used for physical activity and stress relief). Future work in this area of California can begin to parse out rural versus urban greenspaces, as well as the particular types of greenspace such as a crop versus a park or forest that may be differently utilized.

Additionally, this study was likely hindered by some measurement error. Our main exposures of interest—greenspace, air pollution, and neighborhood SES—were based on residential address and not directly measured for each study subject’s entire living environment. Air pollution was measured using distance-weighted averages to monitors near their residences, and greenspace was designated as the density surrounding their residence so we would not have precise measurements of these exposures for a participant who spends most time away from their home or indoors. The census-based neighborhood variables also may not have applied to the participant since only a certain percentage had to be below the poverty level to qualify as a high-poverty neighborhood. However, the neighborhood variables still reflected resources available in the neighborhood and may have correlated with other variables like crime or other stressors, thus still being useful.

A few covariates with potential to confound our observed associations, such as smoking and body mass index (BMI), were unavailable in this dataset. Tobacco smoking has been observed to be inversely associated with preeclampsia [31]. However, the majority of our study population was Hispanic, and previous inquiries in this Hispanic population observed very low levels of smoking. Thus, if we did observe an effect on our estimates due to smoking, we would have expected it to be very small. In a previous study of this population utilizing years where BMI was available, women with preeclampsia were more likely to be obese (BMI ≥ 30) than controls [16]. We performed a sensitivity analysis using the years for which BMI was available and observed the mean BMI to be similar, i.e., between 26.3 and 26.7, across quartiles of residential greenness. Analysis restricted to the subset of data for which BMI was available did not reveal a change in the association observed for the overall dataset between greenspace and preeclampsia. Finally, given that there were roughly 50 statistical tests performed, some of the observed associations may have been due to chance alone.

This study had many strengths. The San Joaquin Valley is a highly polluted area in California and is home to a highly diverse population, thus making it an ideal population to study the interconnectedness of pollution and neighborhood socioeconomic differences. All of the data were acquired using satellite, monitor, census, or health record data, which excluded the possibility of recall bias or other measurement issues related to questionnaires administered directly to participants. This study was also unique given our ability to link multiple California registries and a sample size large enough to allow for stratification by various factors. Given the administrative data, we believe this population to be representative of the population from which they were derived.

## 5. Conclusions

The observed associations between all of our exposures of interest and various preeclampsia phenotypes highlight the importance of this line of inquiry. Specifically, given the heterogeneity observed by neighborhood SES, future research using data on types of greenspace and individual level variables, such as the use of greenspace, physical activity, and stress, is warranted. These variables may help identify what may be driving the inverse associations in higher SES neighborhoods. Such inquiries may inform etiologic and mechanistic investigations, as well as future potential interventions to help reduce the risks of preeclampsia—particularly superimposed preeclampsia on pre-existing hypertension.

## Figures and Tables

**Table 1:** Descriptive characteristics (%) ^a^ of 2282 women with preeclampsia by phenotype and 75,124 controls in California from 2000 to 2006.

	Cases			Controls ^b^
	Mild	Severe	Superimposed	
	*n* = 983	*n* = 1043	*n* = 256	*n* = 75,124
Age (years)				
<20	18.3	17.1	3.5	13.6
20–24	27.4	27.0	16.0	29.7
25–29	21.8	23.6	24.6	27.7
30–34	17.9	17.2	27.0	19.1
≥35	14.6	15.2	28.9	10.0
Missing	0.1	--	--	--
Race/ethnicity				
White, non-Hispanic	29.4	26.1	29.3	30.7
US-born Hispanic	31.7	30.9	25.8	25.4
Foreign-born Hispanic	22.6	25.8	20.7	28.7
Black, non-Hispanic	7.9	6.9	14.8	5.2
Other	7.9	9.7	8.6	9.7
Missing	0.4	0.7	0.8	0.4
Education				
Less than high school	28.0	27.6	25.0	31.2
High school	35.6	34.5	36.7	32.5
More than high school	34.5	35.3	36.3	34.6
Missing	1.9	2.6	2.0	1.7
Parity				
1	53.1	57.2	34.4	35.1
≥2	46.9	42.6	65.6	64.8
Missing	--	0.2	--	<0.1
Payer type for delivery				
Medi-Cal	56.1	52.0	44.9	52.8
Private	41.8	44.4	51.2	44.7
Other	1.8	3.6	3.5	2.4
Missing	0.3	0.1	0.4	0.1
Season of conception				
Winter (Dec–Feb)	26.1	24.5	23.1	26.2
Spring (March–May)	25.9	23.7	23.8	24.9
Summer (June–Aug)	23.7	27.0	23.1	23.6
Fall (Sep–Nov)	24.2	24.8	30.1	25.4
Income below the federal poverty level (proportion greater than 20%) ^c^				
No	55.5	54.0	59.0	57.9
Yes	44.5	46.0	41.0	42.1
Median household annual income (less than $30,000) ^c^				
No	57.4	56.5	60.6	60.2
Yes	42.6	43.5	39.5	39.8

^a^ Percentages may not equal 100 due to rounding. ^b^ Defined as women who delivered in the study period who did not have diabetes (gestational or pre-existing), did not have any hypertensive disorder, and delivered between 37 and 41 weeks. ^c^ From the 2000 US census at the block group level.

**Table 2:** Characteristics of participants (%) ^a^ by Greenspace (average NDVI) in the highest quartile of residential buffers in California from 2000 to 2006.

	100 m Buffer (75th %)	500 m Buffer (75th %)	All Subjects
	*n* = 19,320	*n* = 19,279	*n* = 77,406
Maternal age (years)			
<20	13.6	12.2	13.7
20–24	28.6	27.1	29.6
25–29	26.9	27.8	27.5
30–34	19.6	21.2	19.1
≥35	11.4	11.7	10.2
Maternal race/ethnicity			
White, non-Hispanic	34.3	35.6	30.6
US-born Hispanic	24.2	22.6	25.5
Foreign-born Hispanic	28.6	28.2	28.6
Black, non-Hispanic	4.3	4.1	5.3
Other	8.1	9.0	9.7
Missing	0.5	0.5	0.4
Maternal education			
Less than high school	31.0	28.7	31.1
High school	31.9	31.4	32.6
More than high school	36.0	38.6	34.6
Missing	1.0	1.3	1.7
Parity			
1	36.3	35.7	35.7
≥2	63.7	64.3	64.3
Missing	<0.1	<0.1	<0.1
Payer type for delivery			
Medi-Cal	50.3	46.6	52.8
Private	47.0	50.9	44.7
Other	2.6	2.4	2.4
Missing	<0.1	0.1	0.1
Season of conception			
Winter (Dec–Feb)	26.3	25.8	26.1
Spring (March–May)	24.4	24.7	24.8
Summer (June–Aug)	23.9	24.0	23.7
Fall (Sep–Nov)	25.4	25.5	25.4
Income below the federal poverty level (proportion greater than 20%) ^b^			
No	64.3	70.8	57.9
Yes	35.7	29.2	42.2
Median household annual income (less than $30,000) ^b^			
No	65.9	73.3	60.1
Yes	34.1	26.7	39.9

^a^ Percentages may not equal 100 due to rounding; ^b^ From the 2000 US census at the block group level.

**Table 3:** Adjusted ^a^ associations (odds ratios) between average NDVI within residential buffers, air pollutants, neighborhood SES factors, and preeclampsia phenotypes (*n* = 77,083) ^b^.

	Mild Preeclampsia	Severe Preeclampsia	Superimposed Preeclampsia
	aOR (95% CI)		
100 m Buffer (>75% vs. ≤25%)	0.83 (0.69,1.00)	1.04 (0.87,1.24)	0.86 (0.60,1.22)
500 m Buffer (>75% vs. ≤25%)	0.82 (0.69,0.99)	0.98 (0.82,1.17)	0.56 (0.40,0.80)
CO (>75% vs. ≤75%)	1.06 (0.89,1.25)	1.10 (0.94,1.30)	0.93 (0.67,1.30)
NO_2_ (>75% vs. ≤75%)	1.13 (0.97,1.31)	1.11 (0.96,1.29)	0.99 (0.74,1.34)
PM_10_ (>75% vs. ≤75%)	1.20 (1.04,1.39)	0.99 (0.86,1.15)	1.07 (0.80,1.44)
PM_2.5_ (>75% vs. ≤75%)	1.28 (1.10,1.49)	1.38 (1.19,1.59)	1.23 (0.92,1.65)
Neighborhood Poverty >20% (Yes vs. No)	1.24 (1.08,1.42)	1.31 (1.15,1.50)	1.29 (0.99,1.69)
Median Income <30 K (Yes vs. No)	1.25 (1.09,1.43)	1.29 (1.13,1.47)	1.32 (1.01,1.73)

^a^ Adjusted for maternal age (years), race/ethnicity (non-Hispanic white, U.S.-born Hispanic, foreign- born Hispanic, non-Hispanic Black, Other), season of conception (winter, spring, summer, and fall), parity (1, ≥2). ^b^ Only women without a missing covariate were included in adjusted analyses.

**Table 4:** Associations between greenspace (average NDVI) within 500 m buffer surrounding participant residence (>75% vs. ≤25%) by neighborhood SES factors, 2000–2006.

Preeclampsia Phenotype	Adjusted ^a^ Odds Ratio (95% Confidence Intervals)		*p*-Value Interaction
	High Poverty	Low Poverty	
Mild	0.79 (0.59,1.05)	0.93 (0.73,1.20)	0.44
Severe	1.17 (0.91,1.52)	0.97 (0.75,1.25)	0.27
Superimposed	0.80 (0.45,1.44)	0.46 (0.30,0.71)	0.13
	**Low Income**	**High Income**	
Mild	0.83 (0.62,1.11)	0.91 (0.71,1.16)	0.66
Severe	1.07 (0.82,1.40)	1.06 (0.82,1.36)	0.89
Superimposed	1.01 (0.57,1.81)	0.42 (0.27,0.65)	0.01

^a^ Adjusted for maternal age (years), race/ethnicity (non-Hispanic white, U.S.-born Hispanic, foreign- born Hispanic, non-Hispanic Black, and Other), season of conception (winter, spring, summer, and fall), and parity (1 and ≥2).

**Table 5:** Associations between greenspace (average NDVI) within a 500 m buffer surrounding participant residence by level of exposure to PM_10_ and PM_2.5_, as well as by pollutant and neighborhood SES factors from 2000 to 2006.

Neighborhood SES	Preeclampsia Phenotype	Adjusted ^a^ Odds Ratio (95% Confidence Intervals)		*p*-Value Interaction
		PM_10_ High Exposure	PM_10_ Low Exposure	
Overall	Mild	1.00 (0.69,1.45)	0.81 (0.64,1.01)	0.18
	Severe	1.41 (0.98,2.03)	0.87 (0.71,1.08)	0.01
	Superimposed	0.51 (0.21,1.23)	0.59 (0.39,0.89)	1.00
High Poverty	Mild	0.87 (0.51,1.49)	0.76 (0.52,1.09)	0.52
	Severe	1.45 (0.90,2.35)	1.07 (0.78,1.48)	0.21
	Superimposed	0.54 (0.16,1.84)	1.09 (0.51,2.35)	0.40
Low Poverty	Mild	1.30 (0.75,2.24)	0.90 (0.66,1.22)	0.15
	Severe	1.56 (0.87,2.78)	0.83 (0.62,1.11)	0.02
	Superimposed	0.56 (0.15,2.05)	0.41 (0.25,0.67)	0.54
Low Income	Mild	0.86 (0.49,1.51)	0.84 (0.58,1.21)	0.77
	Severe	1.35 (0.82,2.24)	0.96 (0.69,1.34)	0.19
	Superimposed	0.47 (0.11,2.06)	1.32 (0.65,2.68)	0.27
High Income	Mild	1.28 (0.76,2.17)	0.85 (0.63,1.16)	0.10
	Severe	1.75 (1.00,3.06)	0.90 (0.67,1.21)	0.01
	Superimposed	0.58 (0.19,1.80)	0.40 (0.24,0.66)	0.42
		**PM_2.5_ High Exposure**	**PM_2.5_ Low Exposure**	
Overall	Mild	0.93 (0.65,1.32)	0.84 (0.67,1.05)	0.58
	Severe	1.17 (0.84,1.64)	1.01 (0.81,1.25)	0.28
	Superimposed	0.61 (0.30,1.23)	0.58 (0.38,0.88)	0.69
High Poverty	Mild	1.05 (0.63,1.76)	0.73 (0.51,1.05)	0.21
	Severe	1.41 (0.90,2.21)	1.24 (0.89,1.71)	0.53
	Superimposed	0.86 (0.28,2.63)	0.87 (0.43,1.78)	0.89
Low Poverty	Mild	0.95 (0.57,1.58)	0.97 (0.72,1.31)	0.94
	Severe	1.30 (0.77,2.18)	0.92 (0.69,1.24)	0.17
	Superimposed	0.51 (0.20,1.28)	0.45 (0.27,0.75)	0.65
Low Income	Mild	1.08 (0.64,1.82)	0.77 (0.53,1.12)	0.26
	Severe	0.97 (0.58,1.62)	1.23 (0.88,1.71)	0.62
	Superimposed	0.78 (0.22,2.76)	1.08 (0.55,2.12)	0.76
High Income	Mild	0.96 (0.58,1.58)	0.94 (0.70,1.27)	0.98
	Severe	1.69 (1.03,2.75)	0.96 (0.72,1.29)	0.04
	Superimposed	0.51 (0.22,1.21)	0.43 (0.26,0.72)	0.56

^a^ Adjusted for maternal age (years), race/ethnicity (non-Hispanic white, U.S.-born Hispanic, foreign- born Hispanic, non-Hispanic Black, and Other), season of conception (winter, spring, summer, and fall), and parity (1 and ≥2).

## Data Availability

The data are publicly available from the Office of Statewide Health Planning and Development (OSHPD) with specific approvals from OSHPD and the California Committee for the Protection of Human Subjects.

## Supplementary Materials

The following are available online at https://www.mdpi.com/article/10.3390/ijerph18105127/s1. Table S1: Pearson correlation coefficients of exposures among controls born between 2000 and 2006 in four counties in the San Joaquin Valley of California (*n* = 75,124).
