# Supplementary material for: Greenspace, Air Pollution, Neighborhood Factors, and Preeclampsia in a Population-Based Case-Control Study in California
Source: Int J Environ Res Public Health. 2021 May 12;18(10):5127. doi: 10.3390/ijerph18105127 (PMC8151979; doi:10.3390/ijerph18105127)
Supplement: Supplementary file 1 [file ijerph-18-05127-s001.zip › ijerph-1209469-supplementary.pdf]

**Supplemental Table S1. Pearson correlation coefficients of exposures among controls born between 2000 and 2006 in four counties in the San Joaquin Valley of California (n = 75,124)**									
	100m	500m	CO	NO2	PM10	PM2.5	Poll Score	Poverty	Household income
100m buffer	1.0000								
500m buffer	0.6874	1.0000							
Carbon monoxide (CO)	-0.1027	-0.1336	1.0000						
Nitrogen dioxide (NO2)	-0.0550	-0.1414	0.7530	1.0000					
Particulate matter <10 µm (PM10)	-0.2045	-0.2652	0.5655	0.4867	1.0000				
Particulate matter <2.5 µm (PM2.5)	-0.1147	-0.1602	0.7617	0.5857	0.6994	1.0000			
Pollutant score	-0.1294	-0.1952	0.8089	0.7275	0.7031	0.7861	1.0000		
Proportion of income below poverty level	-0.0719	-0.2073	0.0943	0.1657	0.1730	0.1158	0.1410	1.0000	
Median annual household income	0.0210	0.1821	-0.0761	-0.1609	-0.1655	-0.1197	-0.1327	-0.7993	1.0000
